# Comparable Benefits of Stingless Bee Honey and Caffeic Acid in Mitigating the Negative Effects of Metabolic Syndrome on the Brain

**DOI:** 10.3390/antiox11112154

**Published:** 2022-10-31

**Authors:** Nellysha Namela Muhammad Abdul Kadar, Fairus Ahmad, Seong Lin Teoh, Mohamad Fairuz Yahaya

**Affiliations:** 1 Department of Anatomy, Faculty of Medicine, Universiti Kebangsaan Malaysia Medical Centre, Cheras, Kuala Lumpur 56000, Malaysia; 2 Department of Biomedical Sciences and Therapeutics, Faculty of Medicine and Health Sciences, Universiti Malaysia Sabah, Kota Kinabalu 88400, Malaysia

**Keywords:** honey, high-carbohydrate/high-fructose diet, hippocampus, antioxidant

## Abstract

There is mounting evidence that metabolic syndrome (MetS) contributes to the development of neurodegenerative disorders such as Alzheimer’s disease. Honey, which has been used for generations, is high in antioxidants and has been demonstrated to benefit the brain and mental health by reducing oxidative stress and boosting cognitive outcomes. Honey from the stingless bees of *Heterotrigona itama* has been found to have higher phenolic content compared to other types of honeys. The aim of this study is to investigate the effects of stingless bee honey (SBH) supplementation and to compare it with a pure form of antioxidant, caffeic acid (CA), on MetS parameters and inflammatory markers in the brains of MetS-induced rats. A total of 32 male Wistar rats were divided equally into groups of control, high-carbohydrate high-fructose (HCHF) diet (MetS), HCHF + SBH supplemented (1 g/kg) (SBH), and HCHF + CA supplemented (10 mg/kg) (CA) groups. The total duration for SBH and CA supplementation was eight weeks. The HCHF diet was found to promote hypertension, hyperglycemia, and hypertriglyceridemia, and to increase brain TNF-α levels. Supplementation with SBH and CA significantly reversed (*p* < 0.05) the hyperglycemic and hypertensive effects of the HCHF diet. Although both supplemented groups showed no significant changes to serum HDL or TG, SBH significantly reduced (*p* < 0.05) brain TNF-α levels and increased (*p* < 0.05) brain BDNF levels. Immunohistochemistry investigations of neurogenesis (EdU) and apoptosis (TUNEL) on the cornu Ammonis 1 (CA1) and dentate gyrus (DG) areas of the hippocampus showed no changes with SBH and CA supplementation compared to the control. These findings suggest that SBH and CA have the potential to mitigate HCHF-induced MetS effects and possess neuroprotective abilities.

## 1. Introduction

A rise in the incidence of metabolic syndrome (MetS) is attributed to the globalization of high-calorie diets, which stems from the variety of fast and processed foods that are readily available. As a result, the increase in MetS cases has caused increased morbidity and shorter life expectancy and has been acknowledged as a worldwide public health problem. MetS is a collection of risk factors that include central obesity, insulin resistance, dyslipidemia, and high blood pressure [1]. There is evidence linking MetS with decreased cognitive performance and increased risk of neurodegenerative disorders such as Alzheimer’s disease [2].

MetS can have a negative effect on the brain through three mechanisms: insulin resistance, damage to endothelial blood vessels, and oxidative stress [3]. Type 2 diabetes mellitus (T2DM) has been shown to accompany insulin resistance in the hippocampus [4]. MetS also increases ROS production as a result of increased lipid peroxidation [5]. Chronic micro-inflammatory conditions in MetS involve an increase in immune mediators such as interleukin (IL)-6, IL-1β, and tumor necrosis factor (TNF)-α, and this can contribute to behavioral changes [6].

Current treatment of MetS is more focused on altering the patient’s way of life in order to prevent the possibility of developing the syndrome. This is critical since the pathophysiology of MetS is complex, and the disease processes are still not completely understood. Yet many studies have identified increased reactive oxygen species (ROS), chronic low-grade inflammation, and microvascular damage among important components in the pathogenesis of MetS. Antioxidants are free radical scavengers that can help protect the body from cellular damage. Honey is one of the superfoods that has been identified as having high antioxidant content [7]. Stingless bee honey (SBH), produced by the stingless bees of the *Trigona* spp., has high medical value compared to other types of honey [8]. This honey’s high antioxidant content and low sugar level make it an excellent contender for fighting MetS. SBH generally has higher moisture, ash, water, and free acid content with lower pH and total dissolved solids compared to other honey [9]. Various studies have found that this type of honey possesses antioxidant, immunoregulatory, chemoprotective, anti-atherogenic, and antimicrobial effects on living cells [10]. Meanwhile, caffeic acid (CA) is an antioxidant that can be found in a variety of fruits and vegetables. CA is also a type of phenolic acid found in large amounts in SBH [11]. Phenolic compounds can provide protection from non-communicable diseases through antioxidant properties and their ability to regulate various cellular-level processes at different levels, such as enzyme inhibition, gene expression modification, and protein phosphorylation [12]. Numerous studies have reported CA to be helpful in lowering blood glucose levels, boosting insulin levels [13], and lowering blood pressure [14], all of which can directly prevent the formation of MetS. As a result, CA is an excellent choice for comparison, as we aimed to study the effect of SBH on anthropometrical parameters, MetS parameters, and the molecular structure of rat brains by measuring several markers, such as levels of inflammation, degeneration, and cell plasticity of the brains of MetS-induced rats. CA was used in this experiment as a comparative positive control against SBH.

## 2. Materials and Methods

### 2.1. Animals and Treatment

**Figure 1:**
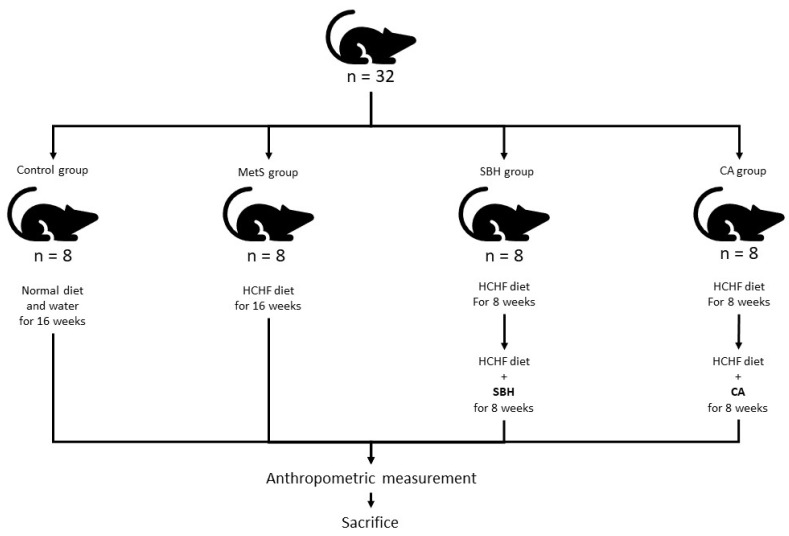
Schematic diagram of groups and treatments: HCHF, high-carbohydrate/high-fructose; SBH, stingless bee honey; CA, caffeic acid.

A total of 32 white male Wistar rats aged 3 months or weighing around 200–250 g, supplied by the Laboratory Animal Research Unit (LARU), Universiti Kebangsaan Malaysia Medical Center (PPUKM), were used for this study. Prior to animal recruitment, ethics approval was obtained from the UKM Animal Ethics Committee (UKMAEC) with approval number ANAT/PP/2018/FAIRUZ/28-NOV./975-NOV.-2018-SEPT-2020. Experimental animals were randomly divided into four groups, including a control group fed with standard rat pellets and tap water ad libitum (Control), while the remaining animals were given a high-carbohydrate/high-fructose (HCHF) special diet and a 25% fructose-based beverage for 8 weeks to induce MetS, followed by 8 weeks of SBH or CA treatment. The HCHF-supplemented rats were then further divided into animals receiving 16 weeks of HCHF diet (MetS group), rats supplemented with 8 weeks of SBH (SBH group), and rats supplemented with 8-weeks of CA (CA group) (Figure 1).

All rats were maintained in separate cages in the Animal Laboratory, Department of Anatomy, with controlled settings such as temperatures between 25–27 °C and 12-h day and night cycles. Rats were acclimatized for at least 7 days in the animal laboratory before any treatment or experiments were carried out. All procedures were carried out in compliance with the UKMAEC guidelines.

### 2.2. Fructose Solution and High-Fructose/High-Fat Diet Preparation

Cornsweet fructose powder (Archer Daniels Midland Company, Chicago, IL, USA) was used to make a 25% fructose solution, which was prepared by dissolving 250 g of the powder in 100 mL of distilled water at room temperature. A total of 155 g of rat pellet powder (Gold Coin Sdn. Bhd., Johor Bahru, Malaysia) was combined with 25 g of Hubbel, Mendel, and Wakeman salt (MP Biomedicals, Santa Ana, CA, USA), 395 g of full-cream sweetened condensed milk, 200 g of melted ghee, 175 g of Cornsweet fructose powder (ADM, Chicago, IL, USA), and 50 g of tap water to make 1 kg of HCHF diet.

### 2.3. Stingless Bee Honey and CA Solution

Pure SBH was obtained from Pusu Jaya Sdn. Bhd. (Sungai Pusu, Selangor, Malaysia) in September 2018. SBH solution was prepared daily with a ratio of 1:1 (SBH: distilled water). It was then stored in glass bottles coated with aluminum foil to block direct sunlight and placed in a refrigerator at 4–8 °C. CA was dosed for the rats at 10 mg/kg [14]. CA solution was prepared by dissolving 100 mg of CA powder into 20 mL of distilled water. Both SBH and CA solution were given for the eight weeks following the HCHF diet via force feeding using a 16 G feeding needle.

### 2.4. Anthropometric Measurement

A measuring tape and weight scales (Nimbus Precision Balance, Adam Equipment, Oxford, CT, USA) were used to measure length, abdominal circumference (AC), and weight of the experimental animals. Blood pressure was measured at the start and end of the study. Initially, rats were confined and allowed to rest for 3–5 min before their tails were fitted with an occlusion cuff that contained the VPR sensor (CODA). A total of three consecutive readings were recorded and analyzed.

### 2.5. Brain Extraction

Twenty-four hours before sacrifice, the rats received an intraperitoneal injection of EdU (Base click, Munich, Germany) solution at a dose of 50 mg/kg, which was used to detect cell proliferation. They were fasted for 12 h and then given a mixture of xylazine and ketamine before sacrifice using a guillotine. Once the brain had been successfully extracted, the hemispheres were randomly divided for either ELISA or immunohistochemistry.

### 2.6. Serum Biochemical Analyses

For biochemical measurements, fasting blood sugar (FBS, Bio Manheim, Shah Alam, Malaysia) and lipid profile (BioAssay Enzyme, Hayward, CA, USA) were measured using the manufacturers’ protocols. Blood samples were collected on the day the rats were sacrificed. After the rats were anesthetized, blood was taken via retro-orbital bleeding using a micro-hematocrit capillary tube into a test tube. The sample was then centrifuged (Eppendorf 5427R, Hamburg, Hamburg, Germany) at 3000× *g* for 5 min at 5 °C. The sample was then sent to the Veterinary Laboratory of the Universiti Putra Malaysia for triglyceride (TG) and high-density lipoprotein (HDL) analyses.

### 2.7. ELISA Test

The brain hemispheres were chosen randomly. Selected brain hemispheres were rinsed with cold phosphate-buffered saline (PBS) x 1 solution and immediately placed in a labeled aluminum foil envelope and stored in a container containing dry ice and ethanol. The samples were stored in a −80 °C freezer before processing. Before being homogenized, the samples were placed at room temperature for 5 min then rinsed with cold PBS. Samples were weighed (100 mg) and homogenized with cold radioimmunoprecipitation assay buffer solution (0.9 mL) (Merck, Darmstadt, Germany) using an iron bead homogenizer at a speed of 5 m/s for 30 s at room temperature four times before being centrifuged (Eppendorf 5427R, Hamburg, Germany) at 12,000× *g* for 5 min at 4 °C [15]. Supernatants were collected into 1.5 mL tubes (100 μL per tube), labeled, and stored at −80 °C until the day of ELISA reading. Rat TNF-α ELISA kits (Elabscience, catalog no. E-EL-R0019), rat IL-6 ELISA kits (Elabscience, catalog no. E-EL-R0015), and rat BDNF ELISA kits (Elabscience, catalog no. E-EL-R1235) were used in this study.

### 2.8. Immunohistology

#### 2.8.1. EdU (5-Ethynyl-2′-deoxyuridine)

The remaining brain hemispheres were fixed with 4% paraformaldehyde (Sigma-Aldrich, Burlington, MA, USA) solution for 24 h at 4 °C and then cryopreserved in 15% and 30% sucrose solution overnight. Subsequently, samples were frozen by snap freezing using an isopentane bath placed on dry ice. The samples were then sliced using a cryostat machine with a thickness of 16 µm. Slides were then rinsed with 3% BSA solution twice. A 0.5% Triton X-100 permeabilization solution was placed over each sample section, and then the samples were incubated for 20 min at room temperature. EdU BCK-IV (Base click, Munich, Germany) was used to detect cell proliferation. A total of 100 μL of the reaction cocktail solution containing deionized water, buffer solution, catalyst solution, and azide dye (6-FAM azide) was spread over each cryosection, followed by incubation for 30 min in the dark. The slides were then examined under a fluorescence microscope under 516 nm emission.

#### 2.8.2. TUNEL Assay

Apoptosis detection kits, POD version 15 (catalog no. 11684817910, Roche Diagnostics GmbH, Mannheim, Germany) containing terminal enzyme solution deoxynucleotidyl transferase from calf thymus and labeled solution were used to detect apoptotic neuronal cells. After the fixation step and washing with PBS, cryosections of rat brain were incubated with a blocking solution (3% H_2_O_2_ in methanol) for 20 min at 15–25 °C. Slides were then rinsed with PBS, incubated with permeabilization solution (0.1% Triton-X-100) on ice for 2 min, and then incubated with the TUNEL reaction solution for 60 min at 37 °C in the dark. The slides were then analyzed under a fluorescence microscope at a detection emission of 515–565 nm.

### 2.9. Statistical Analysis

The data were recorded in the form of mean ± standard error of mean (SEM). Statistical Package for Social Sciences version 20 (SPSS) was used to perform statistical analysis. Data distribution was tested using the Kolmogorov–Smirnov test to determine the normal distribution. Data analyses were done using one-way ANOVA and post-hoc LSD and Tukey tests, while non-normal distribution was analyzed using the Mann--Whitney test. A value of *p* < 0.05 was considered significant.

## 3. Results

### 3.1. Anthropometrical and Physiological Measurements

**Figure 2:**
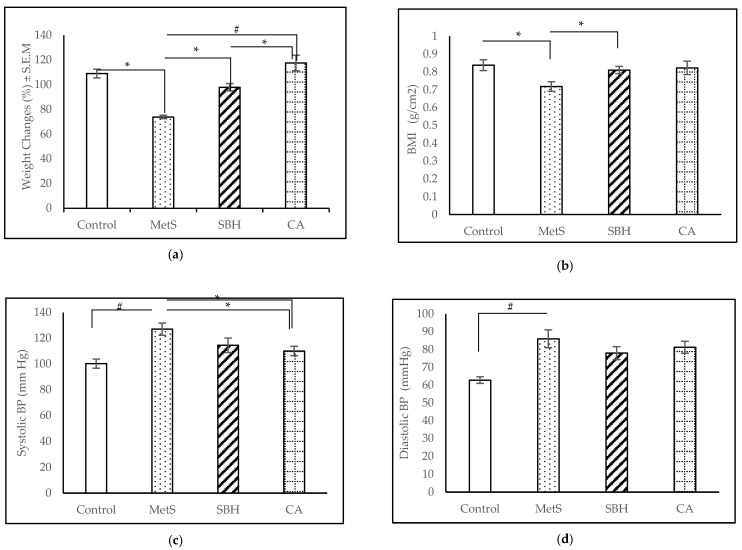
Effects of MetS induction and SBH and CA supplementation on physiological parameters of the experimented rats: changes in (**a**) weight; (**b**) BMI; (**c**) SBP; and (**d**) DBP. Data are presented as mean ± SEM (*n* = 7). * *p* < 0.05; # *p* < 0.001.

Over the course of the experiment, the total body weights of all groups increased gradually (Figure 2a: CA and SBH were supplemented from week 8 until week 16. CA showed the highest weight increase of 117.5 ± 6.19%, followed by the control group with 108.9 ± 3.59%, the SBH group with 97.91 ± 2.96%, and the MetS group with 73.82 ± 1.36%. There were significant differences between MetS and Control (*p* < 0.001), MetS and CA (*p* < 0.001), and SBH compared to CA (*p* = 0.047). AC did not show any significant changes (data not shown), but body mass index (BMI) showed the following results: Control (0.838 ± 0.031 g/cm^2^), MetS (0.718 ± 0.027 g/cm^2^), SBH (0.811 ± 0.021 g/cm^2^), and, finally, CA with (0.823 ± 0.038 g/cm^2^) (Figure 2b). The consumption of the HCHF diet significantly lowered the BMI compared to the control group, whereas supplementation with SBH increased the BMI compared to the MetS group.

Blood pressures were taken via tail cuff. For the SBP readings, the MetS group showed the highest mean systolic blood pressure (SBP) reading (131.11 ± 3.52 mmHg), followed by SBH (118.56 ± 5.26 mmHg), CA (110.00 ± 4.73 mmHg), and Control (99.39 ± 4.39 mmHg) (Figure 2c). There was a significant difference in SBP between the MetS compared to the Control group, with consumption of the HCHF diet increasing SBP (*p* < 0.001). Supplementation with SBH or CA was found to decrease the SBP (*p* < 0.001) against MetS at the end of trial. For diastolic blood pressure (DBP), the MetS group recorded a reading of 86.00 ± 14.34 mmHg, CA (81.24 ± 3.68 mmHg), SBH (77.95 ± 3.90 mmHg), and Control (62.76 ± 2.01 mmHg) (Figure 2d). Analysis of DBP readings between the study groups showed that there was a significant difference (*p* = 0.001) between the Control compared to the MetS group. Supplementation with SBH and CA was found to have no effects on DBP.

### 3.2. Serum Biochemical Analyses

**Figure 3:**
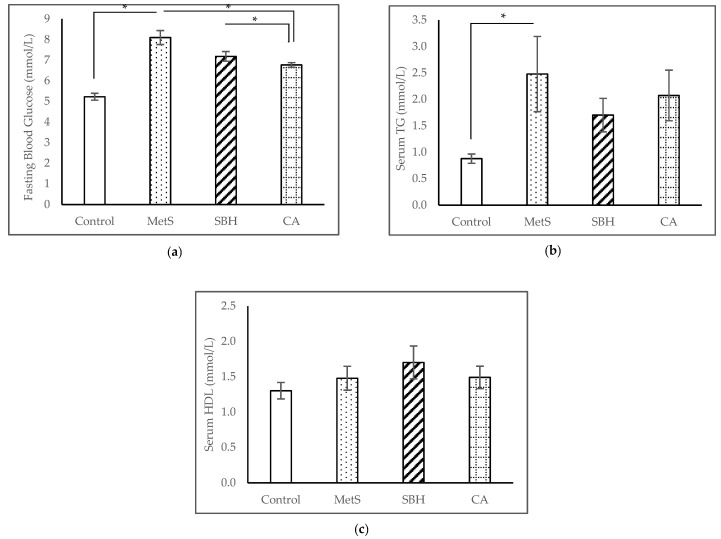
Effects of MetS induction and SBH and CA supplementation on biochemical parameters of the experimented rats: changes in (**a**) FBS; (**b**) serum TG; and (**c**) serum HDL. Data are presented as mean ± SEM (*n* = 7). * *p* < 0.05.

At the end of the experiment, the Control group had the lowest FBS with a mean of 5.23 ± 0.17 mmol/L, followed by CA (6.77 ± 0.11 mmol/L) and SBH (7.19 ± 0.24 mmol/L), while MetS had the highest reading of 8.10 ± 0.34 mmol/L (Figure 3a). The mean FBS readings showed significant differences between the MetS compared to the Control group (*p* < 0.001), whereas supplementation with SBH and CA were able to significantly reduce the FBS against MetS (*p* = 0.001). TG readings (Figure 3b) showed the Control group recorded a mean of 0.88 ± 0.09 mmol/L, MetS (2.48 ± 0.71 mmol/L), SBH (1.70 ± 0.32 mmol/L), and CA (2.08 ± 0.48 mmol/L). Serum TG levels showed significant increases after the HCHF diet compared to the Control group (*p* < 0.001). However, supplementation with SBH or CA was unable to decrease serum TG (*p* > 0.05) following the HCHF diet. The HDL readings (Figure 3c) recorded a mean of 1.85 ± 0.24 mmol/L for the SBH group, CA (1.49 ± 0.16 mmol/L), and MetS (1.40 ± 0.16 mmol/L), and the Control group had the lowest reading of 1.30 ± 0.12 mmol/L. We noticed there were a significant reduction of HDL between Control and MetS group in the eighth week (results not shown here), however, the serum HDL level showed no significant difference between these groups (*p* > 0.05) at the end of the experiment.

### 3.3. ELISA Studies

**Figure 4:**
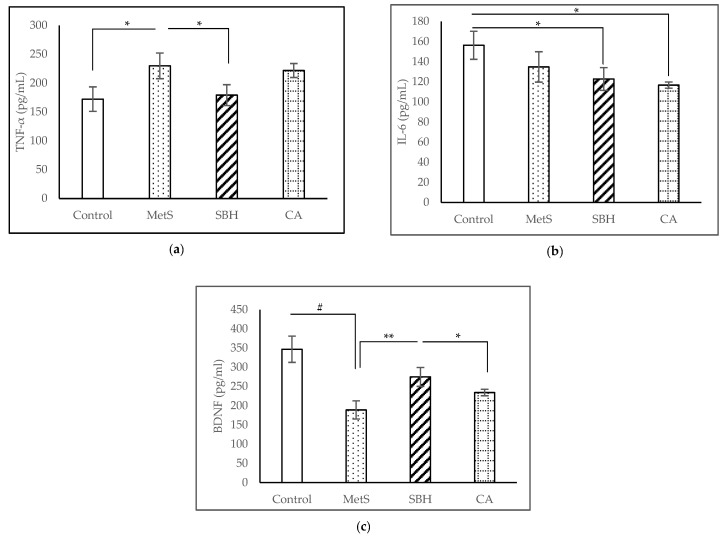
Effects of MetS induction and SBH and CA supplementation on homogenized brain tissue levels of (**a**) TNF-α; (**b**) IL-6; and (**c**) BDNF. Data are presented as mean ± SEM (*n* = 7). * *p* < 0.05; ** *p* < 0.01; # *p* < 0.001.

ELISA tests were carried out to examine the brain inflammatory cytokines TNF-α and IL-6 while examining neuronal survival and growth through BDNF. The TNF-α levels showed (as in Figure 4a) the MetS group had the highest reading of 230.01 ± 22.25 pg/mL, followed by CA (221.89 ± 12.16 pg/mL), SBH (179.28 ± 18.08 pg/mL), and Control with 172.29 ± 21.21 pg/mL. The study showed a significant increase (*p* = 0.033) in TNF-α levels between the MetS compared to the Control (*p* = 0.042). However, only supplementation with SBH, and not CA, was able to reduce the TNF-α level after the HCHF diet (*p* = 0.048).

We found that the level of brain IL-6 (Figure 4b) in Control group recorded a reading of 156.40 ± 13.94 pg/mL, MetS (34.85 ± 15.12 pg/mL), SBH (122.84 ± 11.37 pg/mL), and CA (116.78 ± 3.13 pg/mL). Analysis showed no significant difference (*p* < 0.05) between MetS compared to Control, SBH, and CA. However, there were a significant differences between the Control group compared to SBH (*p* = 0.043) and CA (*p* = 0.01).

ELISA of brain BDNF concentrations (Figure 4c) showed 188.76 ± 23.70 pg/mL for MetS group, followed by CA (234.20 ± 8.47 pg/mL), SBH (274.86 ± 24.54 pg/mL), and Control (346.84 ± 34.19 pg/mL). Analysis showed a significant decrease in BDNF between MetS compared to Control (*p* <0.001) and SBH (*p* = 0.009). CA, however, was significantly decreased compared to Control (*p* = 0.002) and SBH (*p* = 0.038).

### 3.4. Immunohistological Studies

**Figure 5:**
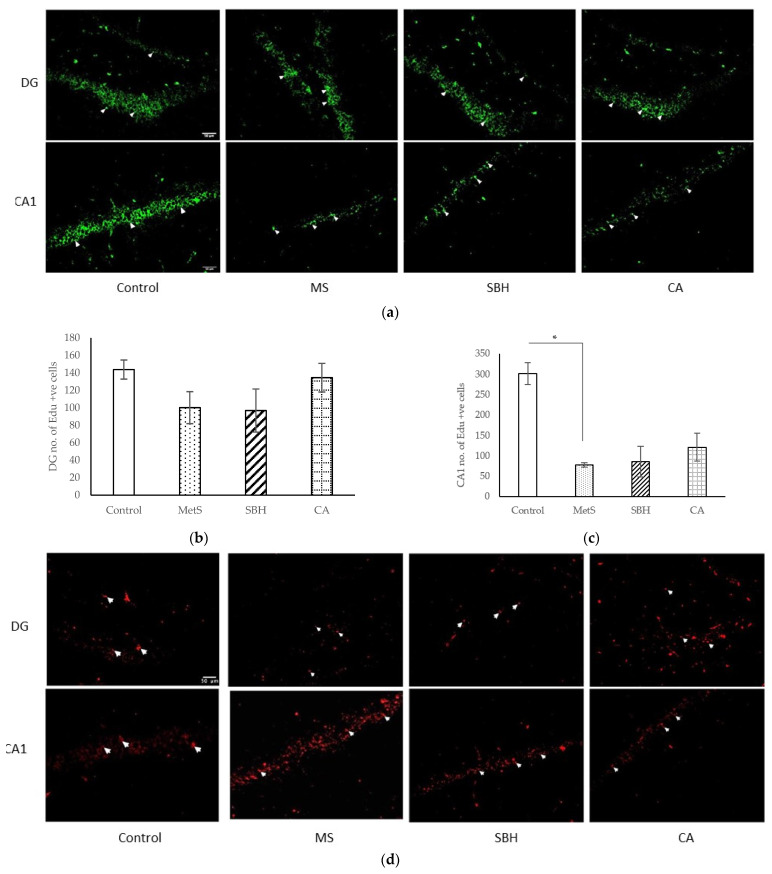

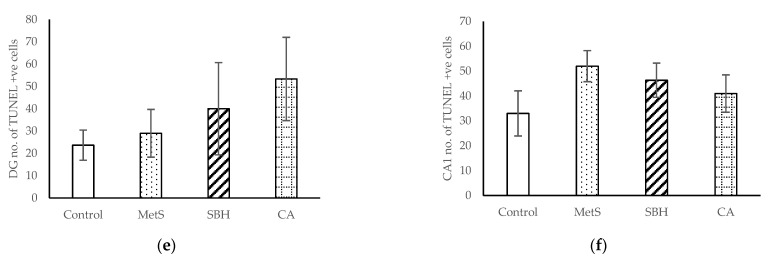
Photomicrographs (**a**,**d**) show the hippocampal areas CA1 and DG, respectively, in the brains of the experimental rats: (**a**) cells undergoing DNA replication tagged by EdU in green (white arrowheads); number of EdU-positive cells in (**b**) DG and (**c**) CA1; (**d**) cells undergoing apoptosis stained by TUNEL are seen as red (white arrowheads); number of TUNEL-positive cells in (**e**) DG and (**f**) CA1. Data are presented as mean ± SEM (*n* = 3). * *p* < 0.05.

Immunohistochemistry was performed on the hippocampal CA1 and the dentate gyrus (DG) of the rat brains. EdU was used to label premature hippocampal neuron cells that signal the process of neurogenesis (Figure 5a). Figure 5b shows the number of EdU-positive cells in the DG region. No significant differences between groups (*p*> 0.05) were observed in this region. In the CA1 region (Figure 5c), the Control group showed the highest number of EdU-positive cells, followed by CA, SBH, and MetS. There were significant differences between the Control group compared to MetS (*p* = 0.046), SBH (*p* < 0.001), and CA (*p* = 0.002). However, there were no significant differences between the MetS, SBH, and CA groups. This study shows that the HCHF diet reduced the number of EdU-positive cells in the CA1 region.

TUNEL assay was used to label cells that underwent apoptosis. Amber-colored positive-label cells were seen on the DG granular layer and the CA1 pyramid layer (white arrows on the photomicrograph indicate positive neuronal cells that have undergone apoptosis) (Figure 5d). Figure 5e shows the number of TUNEL-positive cells in the DG area of the hippocampus, with the CA group showing the most TUNEL-positive cells, followed by SBH, MetS, and Control. No significant differences were found between groups (*p* < 0.05). In the CA1 area (Figure 5f), Control showed the most EdU-positive cells, followed by CA, MetS, and SBH. Similar to findings in the DG area, no significant differences were found between study groups (*p* > 0.05) in the CA1 area.

## 4. Discussion

### 4.1. MetS Induction with HCHF Diet

MetS is a growing epidemic that affects many countries across the globe. As there is no specific treatment that caters to the whole cluster, treatment revolves around its specific diseases. Among the challenges faced by researchers in finding the cure for MetS is finding the best animal model before any treatment can be rendered. Studies inducing MetS using dietary methods aim at mimicking the pathogenesis of MetS in the human population. The HCHF diet proposed by Panchal and Brown [16] is based on the dietary patterns of the Western population. Wong et al. [17], in turn, made some changes to the HCHF diet by replacing beef tallow with ghee, which is more commonly used in the Asian diet. Based on the current results, HCHF diet supplementation for 16 weeks significantly increased the blood pressure (both SBP and DBP), FBS and TG levels in the MetS group compared to the control group, thus fulfilling three of the five MetS criteria. Our findings are in line with the results of our previous study [3].

Obesity was measured in this study by collecting four anthropometric datapoints, such as weight, rat length, and AC, along with BMI calculation. In this study, we found that the MetS-induced group had the lowest percentage of weight gain compared to the other groups. After 16 weeks of the HCHF diet, SBP and DBP levels in the MetS group were significantly higher compared to the control group. For individuals with insulin resistance problems with a negative hyperinsulinemia response, activation of the sympathetic nervous system contributes to vasoconstriction, increased cardiac output, and sodium retention by the renal tubules, which leads to increased blood pressure [18]. In a study by Panchal et al., no significant increase in AC was found, but there was an increase in BMI and weight of mice induced with a high-carbohydrate and high-fat diet together with 25% fructose drinking water for 16 weeks [19]. Another study found that an eight-week high-fructose diet had no effect on BMI or feeding rate, but that it successfully induced insulin resistance in study mice [20]. A similar study by Ramli et al. found that a high-carbohydrate, high-fat diet for eight weeks reduced the body weight of rats, but dual-energy X-ray absorptiometry scan showed that the body fat percentage of mice increased significantly (*p* < 0.05), while the lean body mass decreased (*p* < 0.05) compared to the control group. This suggests that the effect of increasing BMI, AC, and weight is more dependent on dietary carbohydrate constituents than on dietary fructose content [21].

Our study also reported increased TG levels in the MetS group compared to the control. Excessive fructose and lipid intake resulting from the HCHF diet is transported into the bloodstream via lipoproteins such as LDL to adipocytes for storage in the form of TG [21]. This explains the increase in TG levels in the MetS group.

### 4.2. Effect of SBH and CA on MetS Model

This study is a continuation of a previous study of SBH that showed an anxiolytic effect and a protective effect against pyramidal cell damage in the CA1 region of the rat hippocampus on MetS-induced rats [3]. The current study compares the abilities of SBH as an antidiabetic, antihypertensive, and neuroprotective supplement against a well-known antioxidant property exerted by CA. The contents of the SBH used in this study have been thoroughly examined by other groups in our center. Its chemical contents include various types of sugar, protein, minerals, phenolic acid, and flavonoids [22]. LC-MS profiling of SBH from *Heterotrigona itama* found various compounds, including CA, coumaric acid, gluiconic acid, kynurenic acid, acetic acid, pinobanksin, quinic acid, niazimicin, and lanosterol, among others [21]. In contrast to the reduced weight gain in the MetS group, the SBH and CA groups both had percentages of weight gain that were almost the same as that of the control group. This suggests a protective effect of SBH and CA in restoring the weight of MetS-induced mice toward normal. These findings are in line with our previous studies [3]. Both SBH and CA have been shown to possess anti-obesity properties [22,23]. A study by Nasry et al. [24] demonstrated that CA effects weight loss and FBS in MetS-induced mice. CA was found to maintain hypertrophic adipocyte function by increasing the production of adiponectin, which can curb the formation of diabetes, obesity, and cardiovascular disease [25,26]. Meanwhile, SBH inhibits the formation of high-omental fat mass, BMI, and weight gain [27,28].

Additionally, supplementation with SBH and CA were found to significantly reduce SBP readings compared to MetS. However, neither SBH nor CA supplements showed a significant decrease in DBP readings compared to the MetS group. Honey has a high concentration of nitric oxide (NO), a vasodilating agent, which contributes to its antihypertensive properties. Furthermore, the high content of antioxidants in SBH can reduce the effects of ROS while reducing endothelial inflammation, which can increase blood pressure [29]. Previous studies have shown that CA can affect blood pressure via hormonal action and oxidative stress reduction. CA treatment at doses of 5 and 25 mg/kg have been shown to be antihypertensive by increasing serum NO and reducing serum ACE activity in L-NAME hypertensive rats [30]. Furthermore, CA also exerts a protective effect on blood vessel smooth muscle cells. Recent studies have shown that CA is able to inhibit the vasoconstrictive effects of norepinephrine and weaken the effects of angiotensin II, thus providing an effective antihypertensive effect [31].

Supplementation with both SBH and CA showed a significant FBS decreasing effect (*p* < 0.05) compared to the MetS group. This is in accordance with a previous study that reported that SBH supplementation for the last 35 days of 16 weeks of a HCHF diet reduced FBS compared to the control [3]. Additionally, Aziz et al. [32] showed that supplementation with SBH at a dose of 1 g/kg/day for 28 days exerted a protective effect on the pancreatic islet, which can explain the antidiabetic effect of honey. Due to the high carbohydrate content of honey, its efficacy in decreasing plasma glucose is claimed to be debatable. However, combined research supports the anti-diabetic properties of honey. Monosaccharides comprise 75% of the sugar content in honey, with the quantity of fructose exceeding that of glucose [33]. Fructose does not increase plasma glucose and does not require insulin for its metabolism [34].

In regard to the lipid profile, both SBH and CA supplementation showed no effect on the HDL level at the end of the experiment. Our result is in accordance with a previous randomized controlled trial (RCT) by Sadeghi et al. [35], who reported on the consumption of Iranian honey of 50 g/day for eight weeks, and a study by Rashid et al. [36], who used SBH of 30 g for 30 days; they found that honey had no effect on the lipid profiles of subjects with T2DM or a background of IFG. In contrast, another study reported a significant increase in the HDL levels of 40 healthy male subjects in their 20 s who were given a honey supplement (70 g) for four weeks [37]. The results of these RCTs suggest that bee honey and SBH have minimal or no effect on subjects with insulin resistance or DM; instead they can increase the HDL levels of healthy subjects. These findings are in line with the conclusions gathered by Akhbari et al. [38] in their systematic review on the effects of honey intake on adult human subjects.

Both the SBH and CA groups successfully lowered their TG levels, with the SBH group having the lowest TG level. These findings are in line with previous studies. Ramli et al. [28] suggested that the hypotriglyceride effect of SBH is due to the presence of undigestible oligosaccharides such as fructo-oligosaccharide (FOS). FOS in lipid emulsions was found to significantly reduce serum TG by inhibiting the absorption of dietary fat from the small intestine [39]. In an RCT conducted on 370 subjects, honey significantly lowered TG levels [40]. CA can alleviate dyslipidemia by reducing TG and TC, and this can restore hepatic steatosis over the long term [41,42]. Similarly, CA has been shown to provide protection by decreasing TG levels, provide anticoagulation, antioxidant, and anti-inflammatory effects for heart tissue, and lower the expression of mRNA protein-1 chemoattractant TNF-α and monocytes in the kidneys of diet-induced diabetic rats [43].

### 4.3. Effect of SBH and CA on Inflammatory Status and BDNF Level of MetS Model

IL-6 and TNF-α are widely used markers of inflammatory processes. Elevated levels of these two markers often leads to the conclusion that the tissue studied is going through an inflammatory process. In this study, we found that TNF-α was increased but IL-6 was reduced in the brains of MetS-induced rats compared to the control. IL-6 is a proinflammatory cytokine produced by M1 macrophages in normal response to infection and injury. In MetS, adipocyte dysfunction often occurs and is associated with an increased population of M1 macrophages in adipose tissue. This directly increases secretion of IL-6 and other proinflammatory cytokines such as TNF-α. IL-6 has been found to increase in MetS and is associated with central obesity, low HDL, and high TG [44]. However, previous studies have found that IL-6 mRNA has a low expression rate in most brain structures, such as in the hippocampus and cerebellum. Observations have found that IL-6 plays an important role in the differentiation of neuronal cells in these parts of the brain [45]. More and more evidence is now emerging to suggest the role of IL-6 in the prevention of obesity and insulin resistance. IL-6 is a pleiotropic molecule that controls many functions, not only related to inflammation but also to metabolism [46]. IL-6 has also been found to increase the rate of glucose elimination in healthy human bodies [47]. This may explain why there were high quantities of IL-6 in the control group.

In this study, SBH and CA supplementation reduced the TNF-α and IL-6 levels in the brain compared to those of the MetS group. The antioxidant properties of SBH contribute to its anti-inflammatory abilities. Honey is generally rich in antioxidants due to its composition of phenolic acids. A study has shown that giving honey to toxoplasmosis-infected mice decreased TNF-α mRNA expression but maintained IL-6 mRNA expression [48]. These findings suggest the ability of honey as an anti-inflammatory agent with a simultaneous decrease in TNF-α mRNA expression, suggesting once again the need for IL-6 for the normal regulation of the brain. In an in vitro study using lipopolysaccharides as a catalyst for pro-inflammatory cytokine secretion from microphages, the authors proved that SBH can reduce TNF-α secretion by 23% and IL-6 by 43.9% compared to the control group microphage cells [49]. Similarly, CA supplements were found to be able to cross the blood–brain barrier (BBB) and reach brain cells [50]. CA also has been successfully reported to alleviate lipopolysaccharide-induced elevated expression of IL-6 and TNF-α [51].

BDNF in the adult brain has been found to be involved in synaptic transmission processes and synaptic plasticity that aid cognitive function [52]. In this study, brain BDNF levels decreased following 16 weeks HCHF diet supplementation, which is in line with the results of a previous study [53]. Previously, we found that diabetic patients and patients with glycemic disorders had lower BDNF concentrations than healthy subjects [54]. Our study showed that SBH supplementation significantly increased brain BDNF levels compared to the MetS and CA groups but with no significant difference when compared to the control group. This suggests that SBH supplementation can maintain brain BDNF levels at normal levels despite MetS conditions. Previous studies have shown that low doses of SBH can increase BDNF gene expression in mouse brains [55]. It has also been suggested that phenylalanine in SBH may have a memory-enhancing effect, as it can act directly on BDNF to enhance brain and physiological signals [21,55]. Phenylalanine is an amino acid derived from the daily diet that is converted to tyrosine in the body, which is needed in the brain because it is a major constituent of the tyrosine kinase B receptor (TrkB) that acts as a BDNF receptor [56]. BDNF is a metabolic modulator in humans through dietary regulation, increased physical activity, and glucose metabolism [57].

### 4.4. Effect of SBH and CA on Neurogenesis and Apoptosis of Brain Cells of MetS Model

The purpose of immunohistochemical studies performed in this study was to observe the effects of SBH and CA supplementation on neurogenesis (EdU assay) and apoptosis of neurons (TUNEL assay) in the hippocampus. The CA1 hippocampus portion is the area that is the most sensitive to environmental changes, such as hypoxia, toxins, and seizures. DG is the area that undergoes the most rapid neurogenesis [58]. Our study found that neurogenesis in the CA1 region showed significant differences between the control group and the three MetS-induced groups. These findings further prove the CA1 region is sensitive to glucose homeostasis and other molecular changes produced by the HCHF diet. Although neither the SBH nor the CA groups showed a significant increase in neurogenesis cells, the increasing trend in the number of neurogenesis cells suggested that supplementation with CA and SBH could, to some extent, maintain neurogenesis in the hippocampi of MetS-induced rats. Similarly, the control group showed most newborn cells in the DG region. CA and SBH supplementation showed a greater number of neurogenesis cells than the MetS group. This trend further reinforces the effectiveness of CA and SBH supplementation in maintaining the rate of hippocampal neurogenesis of experimental rats. The results of the IHC EdU study on these two hippocampal regions suggest a more optimal effect of CA compared to SBH in maintaining the neurogenesis abilities of the CA1 and DG of MetS-induced mice.

Although the brain is an organ that requires a lot of energy to function optimally, excessive glucose intake leads to process overload [59]. Nutrition-induced insulin resistance significantly affects the insulin signaling pathways within the brain. With that said, the development of brain insulin resistance can contribute to cognitive impairment. Intake of sucrose along with fructose (but not glucose) has been found to reduce the rate of hippocampal neurogenesis by 40% [60]. This explains the lower neurogenesis in the SBH group than in CA group because the presence of sucrose and glucose in honey decreases the rate of neurogenesis to some extent. Additionally, recent studies suggest phenolic acids such as CA can cross the BBB [50,61]. Although SBH cannot cross the BBB, its antioxidant and anti-inflammatory effects on the hippocampal environment through BDNF production and inhibition of proinflammatory elements such as TNF-α can affect neurogenesis and apoptosis of neurons [62,63,64].

The number of apoptotic cells showed no significant increased with MetS induction in either the CA1 or DG regions. Similarly, both the SBH and CA groups showed no effect on the number of apoptotic cells in both regions compared to the MetS group. Our results are in contrast with those of a previous study, where rats fed a high-fat/high-fructose diet had a decrease in the number of hippocampal neurons [65]. However, our previous study on the effect of SBH supplementation on the histological structure of rat hippocampi found that SBH maintained the number of hippocampal pyramidal cells in the CA1 region following a HCHF diet, suggesting a neuroprotective effect of SBH on MetS-related changes in the brains [3]. Another study using a different type of honey, Tualang honey, was also able to improve the pathological changes of the pyramidal cells of the hippocampal CA1 region in the brains of ischemic mice [66]. Ischemia in neurons increases oxidative stress, which catalyzes hippocampal pyramidal cell damage. Glucotoxicity caused by hyperglycemia is a major cause of diabetic neuropathy [67]. Excess electron transport chains and oxidative phosphorylation also lead to mitochondrial dysfunction and oxidative stress [68]. Polyphenols contained in honey have been shown to be antioxidant agents that can protect the brain from oxidative stress damage and reduce inflammation, improving learning potential and cognitive function in brains affected by neurotoxins [69].

## 5. Conclusions

This study demonstrated that an HCHF diet given for 16 weeks significantly increased blood pressure, FBS and TG levels in the MetS group compared to the control group. Furthermore, MetS-induced rats demonstrated significantly increased brain TNF-α levels, while brain BDNF levels and neurogenesis in the hippocampal CA1 region were reduced. Additionally, we also reported the positive effects of SBH from *H. itama* and CA supplementation in alleviating some of these parameters in MetS-induced rats, i.e., reversing the hyperglycemic and hypertensive effects of the HCHF diet. SBH was also able to significantly reduce brain TNF-α levels and increase brain BDNF levels.

## Data Availability

The data presented in this study are available in article.
